# A Social Media Campaign to Promote COVID-19 Vaccination: Cost-Effectiveness Analysis

**DOI:** 10.2196/84540

**Published:** 2026-01-07

**Authors:** Michael William Long, Jeffrey B Bingenheimer, Khadidiatou Ndiaye, Dante Donati, Nandan Rao, Selinam Akaba, Sohail Agha, William Douglas Evans

**Affiliations:** 1 Department of Prevention and Community Health Milken Institute School of Public Health The George Washington University Washington, DC United States; 2 School of Business Columbia University New York, NY United States; 3 Virtual Lab LLC Corvallis, OR United States; 4 Behavioral Insights Lab Seattle, WA United States; 5 Global Health Visions Seattle, WA United States

**Keywords:** COVID-19, vaccination, social media, cost-effectiveness, health promotion

## Abstract

**Background:**

Vaccine hesitancy has increased in recent decades internationally, which sets up a critical barrier to the rapid deployment of novel vaccines against infection with SARS-CoV-2.

**Objective:**

This study used a quasi-experimental design to evaluate the cost-effectiveness of a social media intervention to reduce COVID-19 vaccine hesitancy implemented in Nigeria in 2022.

**Methods:**

The intervention targeted health care providers and adults from the general population who were users of a specific social media platform. We used published estimates from a quasi-experimental evaluation of the campaign’s effectiveness compared to the status quo across 6 intervention states and 31 comparison states over a 10-month period. We estimated the cost-effectiveness of the campaign in terms of cost (2022 US dollars) per person vaccinated using a decision tree analysis and probabilistic sensitivity analysis.

**Results:**

On the basis of the quasi-experimental trial, the campaign led to a crude 6.4–percentage point increase (219/692, 31.6% vs 117/463, 25.3%; *P*=.045) in vaccination rates and an adjusted 7.8–percentage point increase (95% CI 1.68-14.2; *P*=.02) controlling for age group, gender, educational level, religion, and occupation among the 20% (1933/9607) of the overall sample who were unvaccinated and in the persuadable middle. Scaled to the overall population, the campaign led to a 1.57–percentage point (95% CI 0.337-2.87; *P*=.02) increase in the proportion of those vaccinated against COVID-19 among those reached by the social media campaign. The social media campaign resulted in 58.3 million impressions and 1.87 million people reached for a total societal cost of US $1.15 million, or US $0.61 per person reached. This resulted in an incremental cost-effectiveness ratio of US $54.70 (95% uncertainty interval US $20.90-$163) per person vaccinated.

**Conclusions:**

A social media–based campaign to address COVID-19 vaccine hesitancy in 6 states in Nigeria resulted in an increase in vaccination rates. The cost-effectiveness of the campaign compared to no campaign is comparable to that of other campaigns promoting COVID-19 vaccine uptake. The cost per person vaccinated due to the social media campaign was 1% to 8% of the estimated cost per life year saved by vaccination against COVID-19 in low- and middle-income countries. Investing in social media campaigns would likely be a cost-effective approach to increase vaccine uptake and save lives.

## Introduction

The COVID-19 pandemic led to the death of 15 to 20 million people worldwide up to 2021 [1,2]. In response to this threat, governments and private companies demonstrated high capacity for innovation; the rapid development and testing of multiple effective vaccines stands out as a critical success [3]. The pandemic also highlighted ongoing systemic failures in global and national public health systems, including limited capacity for surveillance, communication, and distribution of preventive materials and services [4]. These failures exacerbated existing health inequities within and between countries.

The potential impact of the successful development, manufacture, and distribution of effective vaccines was not fully realized due to the public health system’s inability to communicate the safety and benefit of the new vaccines in the context of widespread mis- and disinformation about the pandemic and the public health response. Building on well-established antivaccine movements, COVID-19 vaccine hesitancy emerged as a major barrier to the control of the pandemic [5]. By November 2023, a total of 80% of people living in high-income countries had received at least one dose of a COVID-19 vaccine compared to 33% of people living in low-income countries [6]. In the years before the COVID-19 pandemic, researchers were evaluating the potential use of social media communication campaigns to address vaccine misinformation and increase vaccine uptake. Previous vaccine promotion campaigns addressing vaccine hesitancy have mostly targeted a narrow set of vaccines (eg, influenza and human papillomavirus in high-income countries and diphtheria, tetanus, pertussis, and polio in middle- and low-income countries) [7]. Reviews of health promotion campaigns covering communicable and noncommunicable diseases on social media have found limited or mixed evidence of reported or observed behavior changes (ie, high engagement) and more reports of interaction with posts or changes in knowledge and attitudes (ie, low to medium engagement) [8,9].

With this promising but mixed and limited research base, and accompanied by calls for development of theoretically based and practice-based social marketing strategies [10], funders and public health organizations rapidly implemented social media campaigns to promote COVID-19 vaccine uptake. Initial evaluations of efforts to promote COVID-19 vaccination or other disease control behaviors through social media campaigns have been positive but with low to moderate effects, leading the public health community to consider whether and how to invest in a sustainable public health social media communication infrastructure [11-14]. Social media campaigns have the potential to reach targeted audiences with tailored messages in ways that may improve both impact and efficiency compared to mass media campaigns [15].

We evaluated the cost-effectiveness of a targeted social media campaign to promote vaccination against COVID-19 among health care providers and other adults in their social environment in Nigeria in 2022. By May 2022, after recording 250,000 COVID-19 cases, Nigeria had received enough COVID-19 vaccines to cover 25% of the population and had administered the first dose to 13% and the second dose to 8% of the population [16]. High levels of vaccine acceptance (76%) in late 2020 were being reported to be much lower as more data were published in 2021 (40%-60%) [16,17]. The World Bank, which classifies Nigeria as a lower-middle–income country, reported that 38% of the Nigerian population accessed the internet in 2022 [18]. A rapid rise in the use of social media in Nigeria and its complex role in the response to COVID-19 had been reported by the time the social media campaign in this paper had been implemented [19].

In this analysis, we aimed to evaluate the cost of implementing a social influencer–based social media campaign and estimate the value of the campaign in terms of cost per person vaccinated, which can be compared to other campaigns targeting vaccine uptake.

## Methods

### Overview

The prospective economic analysis plan was included in the overall analysis plan submitted to the funder and has not been published elsewhere. This project followed the guidelines of the Second Panel on Cost-Effectiveness in Health and Medicine and the reporting guidelines from the Consolidated Health Economic Evaluation Reporting Standards checklist [20,21]. The data used in the model synthesis were collected from 2021 to 2022. The analysis was completed in 2023.

### Intervention Description

This cost-effectiveness analysis is based on the implementation and quasi-experimental evaluation of a 10-month social media campaign promoting vaccination against COVID-19 in Nigeria among health care workers and those in their social networks in 2022 [22]. The campaign was designed and implemented by a team of designers and local organizations and delivered through Facebook and Instagram. The campaign included provaccination social norms and vaccine hesitancy reduction messages delivered by social influencers (eg, local celebrities, health care providers, and religious and business leaders). The campaign theory of change was based on the theory of diffusion of innovations; social norms theory; and the motivation, opportunity, and ability framework [23-25].

### Study Population and Setting

The intervention was implemented in 6 states in Nigeria (Anambra, Bauchi, Lagos, Niger, Rivers, and Sokoto), with participants in the control condition recruited from the Federal Capital Territory and all other states. Participants were eligible if they were aged ≥18 years, had a Facebook account registered in Nigeria and received recruitment advertising in their live feed promoting a study on COVID-19 vaccination, had not been previously vaccinated against COVID-19, and were defined as members of the “persuadable middle” [22]. Those who responded “Definitely” or “Definitely not” to the question “Would you take a COVID-19 vaccine that is approved for use in Nigeria if offered to you?” were excluded based on not being in the persuadable middle. While people in low- and middle-income countries (LMICs) generally have higher vaccine acceptance than those in high-income countries. Nigeria faced vaccine availability and other challenges that may have impacted vaccine hesitancy differently than in higher-income settings, including perceptions that safety and efficacy had not been adequately evaluated in that setting [26-28].

### Cost Evaluation

We used the standard microcosting approach, for which we evaluated all component costs of the intervention instead of using a global project budget. Microcosting includes 3 main steps: identification, measurement, and valuation. To identify the resources used, we prospectively developed a detailed description of the intervention activities and identified necessary resources for each activity. Resources were measured and valued using actual reported expenditures from implementing partners and reported or estimated opportunity costs for the nonbudgeted time from implementing partners, influencer organizations, and participants. Direct costs were all reported in US dollars by the implementing partners and were adjusted for inflation to 2022 US dollars. Opportunity costs accrued in Nigeria were estimated in 2022 Nigerian naira. Nigerian currency was converted to purchasing power parities, with total costs reported in 2022 purchasing power parities, which is equivalent to 2022 US dollars. Costs were converted in 2023. As we did not assess health or economic benefits of vaccination, we did not include opportunity costs of individuals or direct health care sector costs for receipt of the vaccine.

### Intervention Reach

The intervention included 245 distinct advertising campaigns implemented on the Facebook social media platform, which means that the campaigns may have included distinct creative content or audience-targeting and promotion methods and their unique individual reach could not be combined with that of other campaigns. For each of these campaigns, the platform reported the total number of unique individuals receiving campaign messages (reach), the total impressions (ie, the number of times the campaign message was displayed on the target audience member’s screen), and a range of engagement metrics for each of these campaigns. Because we did not have access to the total number of unique individuals reached across all campaigns, we estimated reach based on the largest reported reach across all campaigns. Due to a lack of data on the degree of overlap within a targeted campaign, we based our reach estimate on a conservative assumption that there was complete audience overlap across campaigns.

### Cost-Effectiveness Analysis

We used a societal and payer perspective, which captured both the budgetary costs of implementing a similar campaign in the future and the opportunity costs of implementing partners and individuals engaging with campaign messages. The comparator was the status quo (ie, the current state of affairs in the absence of this intervention), which was chosen based on the intervention design and effect estimate. The time horizon for this study was 1 year to capture planning and implementation; we did not have the capacity to model longer-term health and cost effects following a change in vaccination rates. We did not discount costs or benefits over the 1-year time horizon.

### Outcome Measurement

The primary outcome for this study was vaccination against COVID-19. The incremental effect of exposure to the advertising campaign was estimated from a survey of 10,965 participants who were users of the Facebook social media platform. Of the initial 10,965 participants screened for eligibility, 6198 (56.5%) were excluded as already vaccinated, 1476 (13.5%) were excluded for not being in the persuadable middle, 675 (6.2%) were excluded for missing baseline data, 648 (5.9%) were excluded for not meeting the age criteria, and 35 (0.3%) were excluded for having a duplicate ID. The remaining 17.6% (1933/10,965) of the participants were enrolled in the study. Surveys were fielded to the same cohort, with baseline data collection taking place during the period from December 1 to 31, 2021; first follow-up data collection taking place during the period from March 1, 2022, to April 30, 2022; and second follow-up data collection taking place during the period from October 1 to 4, 2022. Of the 1933 participants enrolled in the study, 1155 (59.8%) completed the first follow-up, and 462 (23.9%) completed the second follow-up. Exposure was based on state of residence, with the intervention implemented in 6 states (Anambra, Bauchi, Lagos, Niger, Rivers, and Sokoto) and control participants recruited from all other states in Nigeria.

Participants were recruited through a social media–based research platform called Virtual Lab. Recruitment was stratified by whether participants were health care providers, with the goal of recruiting 50% of the sample from the health care provider community. COVID-19 vaccination uptake was measured through a single question: “Have you received a COVID-19 vaccine?” Participants could respond as follows: “Yes, a single-dose vaccine”; “Yes, the first dose of a two-dose regimen”; “Yes, both doses of a two-dose regimen”; and “No.” Due to changes in the types of vaccines available, as well as recommendations for boosters, we collapsed the outcome into a binary “vaccinated or not vaccinated” outcome.

The effect of the intervention was estimated using a linear regression model predicting vaccination status at the midpoint and final survey. The primary independent variable in each model was exposure to the intervention. Adjusted models included the following control variables: age group, gender, educational level, religion, and occupation. We used clustered SEs to account for nesting within state of residence. Additional details on the evaluation of the intervention on vaccine uptake are reported elsewhere [22].

For the purposes of this cost-effectiveness analysis, we estimated the reach of the campaign in the intervention states based on the impressions reported by the Facebook social media platform. Impressions are defined as an individual user’s exposure to specific content on the platform that may or may not result in active engagement, such as liking, commenting, or following the account that disseminated or originated the content [29]. Impressions have been shown to account for most of the information exposure on social media, have low correlation with active engagement or “expression,” and be independently correlated with user-reported influence of a given information source [29].

### Uncertainty Analyses

We conducted a probabilistic sensitivity analysis by sampling from the distributions of all parameters with measured uncertainty (Table 1). We included the following scenario analysis: instead of using the effect estimate from the first follow-up from the original outcome study [22], we used the effect estimate from the second follow-up period from the same study. We did not evaluate the heterogeneity of the intervention effect or distributional effects of the intervention. Decision tree models and the probabilistic sensitivity analysis were conducted using TreeAge Pro (R2.0; TreeAge Software, LLC).

**Table 1:** Summary of inputs for the cost-effectiveness analysis of the COVID-19 vaccine promotion social media campaign in Nigeria in 2022.

Variable			Source	Point estimate (95% uncertainty interval)		Distribution (parameters)
Target population already vaccinated at the start of the campaign (%)			Quasi-experimental trial data [22]	64.5 (63.5 to 65.5)		Binomial (p^a^=0.645, n^a^=9607)
Persuadable middle population among those unvaccinated (%)			Quasi-experimental trial data [22]	56.7 (55.1 to 58.3)		Binomial (p=0.567, n=3409)
Percentage point increase in vaccination status due to treatment among the persuadable middle			Quasi-experimental trial data [22]	7.8 (1.68 to 14.2)		Normal (mean 0.078, SD 0.032)
Campaign reach			Meta advertiser platform	1,870,000		—^b^
Average engagement time per media impression (s)			Publisher analysis [30]	1.7		—
Total campaign impressions			Meta advertiser platform	58,300,000		—
Total cost (US $)			Campaign microcosting	1,150,000		—
Cost per person reached (US $)			Calculation	0.613		—
**Sensitivity and scenario analyses**						
	Scenario 1: percentage point increase in vaccination status due to treatment among the persuadable middle using the second follow-up	Quasi-experimental trial data [22]		11.0 (−0.00337 to 0.225)	Normal (mean 0.110, SD 0.058)	

^a^Parameters of each named distribution, where p denotes the probability and n denotes the number of trials.

^b^Not applicable.

### Ethical Considerations

All procedures performed in studies involving human participants were in accordance with the ethical standards of the institutional or national research committee and with the 1964 Declaration of Helsinki and its later amendments or comparable ethical standards. This evaluation was approved by the George Washington University Institutional Review Board (NCR213708), as well as by the National Health Research Ethics Committee in Nigeria (NHREC/01/01/2007-04/10/2021). No identifiable data were used in this study. All participants provided informed consent to participate in the study following the institutional review board–approved protocol. Participants were compensated with 400 naira (approximately US $1) for completion of the 40-item survey implemented through the Facebook Messenger chat function.

## Results

The intervention generated 58,255,000 total impressions across 245 distinct advertising campaigns, which, on the Meta platform (the company that owns Facebook), included one or more sets of individual advertisements. Distinct campaigns were run to allow the intervention to best measure and optimize performance against advertising objectives. The mean reach (unique individuals generating one or more impressions) per campaign was 100,000 (SD 176,000; range 1000-1,873,000). On the basis of an assumption that there was complete overlap across distinct advertising campaigns, the intervention reached 1,873,000 unique individuals.

We summarize intervention costs by activity category in Table 2. Due to the use of marketing labor in the United States and the United Kingdom as well as dollar-denominated contracts with partners in Nigeria, the payer costs accounted for 93% of the total societal costs even though the paid hours to implement the project constituted 14% of the total person-time included in the societal perspective.

**Table 2:** Cost of the COVID-19 vaccine promotion social media campaign by activity in Nigeria in 2022.^a^

	Payer perspective (US $)	Societal perspective (US $)
Government liaison	73,400	73,400
Monitoring and evaluation	98,300	98,300
Campaign development	360,000	360,000
Advertising expenditure	102,000	102,000
Advertising campaign implementation	134,000	134,000
Stakeholder management	293,000	293,000
Participant engagement with advertising	—^b^	77,700
Influencer campaign implementation	—	7520
Total	1,060,000	1,150,000

^a^Costs may not add up due to rounding.

^b^There are no participant opportunity costs included in the payer perspective.

Across both the control and intervention samples (excluding those who were ineligible based on age, duplicate ID, and missing baseline data), 64.5% (6198/9607) of the participants were already vaccinated at baseline. The vaccination rate among this sample of Facebook users was substantially higher than the 13% single-dose uptake reported at a similar point in the rollout (eg, May 2022) [16]. Of the 3409 participants screened in the study who were not vaccinated and were otherwise eligible, 1933 (56.7%) were considered to be in the persuadable middle and were enrolled in the study. In a previous study, we estimated that the intervention led to a 7.8–percentage point increase (95% CI 1.68-14.2) in vaccine uptake controlling for demographic variables among those in the persuadable middle.

In the primary analysis, we estimated that the incremental cost of the intervention per person reached was US $0.63 and the incremental percentage point increase in vaccination prevalence was 0.0157 (95% uncertainty interval [UI] 0.00337-0.0287). This resulted in an incremental cost-effectiveness ratio of US $54.70 (95% UI US $20.90-$163), which means that it cost US $54.70 more than the status quo (ie, the current state of affairs without the intervention) for every additional vaccination.

In scenario analysis 1, we used the effect estimate from the second follow-up of the same study as the primary analysis. In this scenario, the larger percentage point increase in vaccinations per person (0.0221 vs 0.0157) than in the no-intervention condition reduced the incremental cost-effectiveness ratio almost by half (US $29.60, 95% UI negative to US $180; Table 3). The UI includes 0 due to the smaller sample at the second follow-up and resulting marginally significant coefficient reported in the evaluation study. We found that using this estimate resulted in 3% of all model iterations having a negative effect.

**Table 3:** Cost-effectiveness results of the COVID-19 vaccine promotion social media campaign in Nigeria in 2022.

	Mean (95% uncertainty interval)
Incremental cost per person reached (US $)	0.613 (0.613 to 0.613)
Incremental increase in COVID-19 vaccinations per person exposed to the campaign	0.0157 (0.00337 to 0.0287)
Incremental cost-effectiveness ratio (US $ per vaccination)	54.70 (20.90 to 163)
Scenario 1: incremental COVID-19 vaccination per person	0.0221 (−0.000649 to 0.0452)^a^
Scenario 1: incremental cost-effectiveness ratio (US $ per vaccination)	29.60 (negative to 180)^b^

^a^For scenario 1, we used an alternative estimate of the effectiveness of the intervention from the second follow-up period of the same intervention used for the primary analysis.

^b^A total of 3% of the model iterations were negative.

## Discussion

### Principal Findings

In this cost-effectiveness analysis of a social media campaign promoting vaccination against COVID-19 among health care workers and adults in their social environment in Nigeria in 2022, we found that the intervention increased vaccination rates among the target audience at a cost in line with similar efforts in the field.

Incremental cost-effectiveness estimates of media campaigns promoting vaccine uptake vary substantially. On the basis of an analysis of attitude changes as a result of social media campaigns run by 174 public health organizations during the COVID-19 pandemic and another study linking attitudes to vaccination outcomes, Athey et al [31] estimated that the campaigns cost US $5.68 per person vaccinated. The study by Athey et al [31] only incorporated the cost of advertising, which accounted for only 12% of the total costs of running and participating in the campaign in our study. This suggests that our estimate of US $54.70 is likely consistent with that of the analysis by Athey et al [31] (which estimated that it would cost US $48 per person vaccinated assuming a similar cost structure) and highlights the importance of incorporating as many relevant costs as feasible when presenting the cost-effectiveness of social media campaigns.

Because there is no willingness-to-pay threshold for the cost of an incremental person vaccinated, it may be useful to integrate the findings of this study with those of others that have measured the cost per year of life saved (YLS) or cost per disability-adjusted or quality-adjusted life year. A study estimating health benefits and donor costs of increase in COVID-19 vaccination rates in 91 LMICs found that spending on vaccination would cost between US $670 per YLS and US $7820 per YLS depending on the level of vaccination achieved [32]. The authors noted that the cost per YLS for COVID-19 vaccination was similar to the cost for antiretroviral therapy for HIV under the President’s Emergency Plan for AIDS Relief, which they estimated at US $4310 per YLS using the total budget and life years saved from the President’s Emergency Plan for AIDS Relief 2004 to 2013 [31]. The cost per person vaccinated in this study (US $54.70) was between 1% and 8% of the estimated cost per YLS by vaccination against COVID-19 in the 91 LMICs in the aforementioned study [32]. To further contextualize the value of the social media campaign evaluated in this study, vaccination against COVID-19 in LMICs was estimated to prevent 20.39 deaths per 10,000 people vaccinated; each death from COVID-19 was separately estimated to lead to 16 years of life lost [33,34]. This means that, for each person vaccinated, there was an average of 0.0326 (20.39 × 16/10,000) years of life lost prevented. On the basis of the estimates of the variable cost of vaccination delivery after rollout of a national campaign (US $10 for the vaccine and US $2.46 for delivery) and the cost of promotion obtained from this study (US $54.70), the marginal cost for each vaccination delivered would be US $67.16, leading to an estimate of US $2060 per year of life lost averted. The value of rapidly disseminating science-based vaccine promotion in terms of within-country health benefits likely underestimates the benefits of responding to shared global vulnerabilities with shared investments in mutually beneficial solutions such as vaccination. Baker et al [35] highlight this need for rapid collaboration as they paint an alarming picture of our new era of globally shared infectious disease risk caused by the confluence of climate change, urbanization, migration, travel, and intensifying trade of plants and animals.

Much of the work to prepare and launch this specific campaign to increase COVID-19 vaccine uptake could support other public health communications campaigns in Nigeria and potentially other countries. Moving the intervention to scale, such as all 37 states instead of the 6 in the intervention arm of this study, would spread fixed costs across a much larger population and reduce the cost per person vaccinated substantially. Goulbourne and Yanovitzky [36] argue that the COVID-19 pandemic clarified the role of health communication infrastructure as a social determinant of health and that public health organizations will need to invest in hyperlocal health communication capacity across populations to address health inequities. They suggest that training and providing ongoing technical support to trusted intermediaries is one approach to providing hyperlocal health communication at scale. The intervention evaluated in this study did implement the COVID-19 vaccine promotion social media campaign through 12 local health organizations and 10 other local influencers. The involvement of local influencers to shape and deliver health messages was considered an essential component of the campaign. This approach could limit the degree to which the intervention could be scaled at a lower marginal cost.

A primary limitation of this cost-effectiveness analysis is that we were not able to obtain a specific estimate of the total unique individuals reached by the intervention on the Meta platform. To be conservative, we estimated a total intervention reach of 1.87 million unique users based on the reach of the largest single campaign and not the 24.5 million reached if we summed the reported reach estimates for all campaigns. Our estimated US $0.61 per person reached by the campaign would instead be US $0.05, shifting the cost per vaccination from US $54.70 to US $2.98. This order of magnitude difference in the cost-effectiveness of the intervention emphasizes the importance of understanding how social media reach metrics are reported and how studies estimating the same cost-effectiveness outcomes (eg, cost per person vaccinated against COVID-19) are using these metrics. The lack of comparability across studies may be further compounded when studies only use active engagement or expression as a measure of campaign reach [29].

The extent of competing social media and other communication campaigns promoting vaccination against COVID-19, as well as the high levels of mis- and disinformation about the pandemic and the vaccines on the same social media platforms, created another limitation. The incremental effect of the intervention campaigns on the message environment was lower than it would have been in a nonpandemic context. We were not able to assess any competing or synergistic effects of the campaign due to variation in individual or community media environments, nor were we able to evaluate how the campaign interacted with other public health campaigns on the same platform or across channels. Extrapolation of findings from this study period to future pandemics may be limited by the rapidly changing nature of the social media landscape, including as it relates to platform responsibility to address public health misinformation. The recent divergence in the degree of regulatory control over content moderation between the European Union’s Digital Services Act requirement that platforms address the systemic risks posed by misinformation and American jurisprudence’s strengthening of free speech protections of content moderation means that mostly American corporations will potentially pursue jurisdictionally fragmented approaches to misinformation during the next pandemic [37].

We used a self-reported measure of vaccination, which could potentially overestimate the effect of the intervention. Stephenson et al [38] reported that, among a sample of approximately 2000 patients with both self-reported and recorded COVID-19 vaccination status in a hospital setting, the self-reported and recorded vaccination status matched for 95% of the participants. While we used existing studies on the cost-effectiveness of vaccination in similar settings [32], we did not directly estimate how the campaign affected health outcomes, which may vary based on, among other factors, the vaccination level in the community, underlying demography and health status of the population, type of vaccine used, and health care system cost and effectiveness. Incorporating these factors within evaluation of new health communication and other strategies is likely infeasible for most interventions but could be accomplished by partnering with modeling groups that do address these factors or through sustained support of modeling consortia that could share modeling capacity more rapidly during future pandemics [39].

### Conclusions

We found that a local influencer–based social media campaign implemented in 6 states in Nigeria during the COVID-19 pandemic increased COVID-19 vaccination rates among those exposed to the campaign. The campaign demonstrated comparable cost-effectiveness to that of other COVID-19 vaccination campaigns when accounting for differences in cost data included across studies. When combined with existing estimates of the effect of vaccination against COVID-19 on mortality and years of life lost per death due to COVID-19, this intervention achieved a lower cost per year of life lost averted (US $2060) than debated but recognized thresholds of 3 times the national gross domestic product per year of life lost averted [40]. Boosting the reach of vaccination efforts through influencer-based social media campaigns such as the one implemented in this study is likely to be a cost-effective approach to save lives.

## Abbreviations

LMIC: low- or middle-income country
UI: uncertainty interval
YLS: year of life saved

## Data Availability

The datasets generated or analyzed during this study are available from the corresponding author on reasonable request.
